# Gingival Crevicular Placental Alkaline Phosphatase Is an Early Pregnancy Biomarker for Pre-Eclampsia

**DOI:** 10.3390/diagnostics11040661

**Published:** 2021-04-07

**Authors:** Alejandra Chaparro, Maximiliano Monckeberg, Ornella Realini, Marcela Hernández, Fernanda Param, Daniela Albers, Valeria Ramírez, Juan Pedro Kusanovic, Roberto Romero, Gregory Rice, Sebastián E. Illanes

**Affiliations:** 1 Centre for Biomedical and Innovation Research, Department of Periodontology, Faculty of Dentistry, Universidad de Los Andes, Santiago 7620001, Chile; or.realini@gmail.com (O.R.); fernandaparam@gmail.com (F.P.); 2 Department of Obstetrics and Gynecology, Faculty of Medicine, Universidad de Los Andes, Santiago 7620001, Chile; maxmonck@gmail.com (M.M.); sillanes@uandes.cl (S.E.I.); 3 Laboratory of Periodontal Biology, Department of Pathology, Faculty of Dentistry, Universidad de Chile, Santiago 8330015, Chile; mhernandezrios@gmail.com; 4 Department of Statistics, Faculty of Dentistry, Universidad Mayor, Santiago 8580745, Chile; daniela.albers@gmail.com; 5 Department of Public Health and Epidemiology, Faculty of Dentistry, Universidad de los Andes, Santiago 7620001, Chile; vramirez@uandes.cl; 6 Department of Obstetrics and Gynecology, Hospital Sótero del Río, Santiago 13201, Chile; jkusanovic@med.puc.cl; 7 Division of Obstetrics and Gynecology, Faculty of Medicine, Pontificia Universidad Católica de Chile, Santiago 8331150, Chile; 8 Perinatology Research Branch, NICHD/NIH/DHHS, Detroit, MI 48201, USA; prbchiefstaff@med.wayne.edu; 9 Department of Obstetrics and Gynecology, University of Michigan, Ann Arbor, MI 48109, USA; 10 Department of Epidemiology and Biostatistics, Michigan State University, East Lansing, MI 48824, USA; 11 Center for Molecular Medicine and Genetics, Wayne State University, Detroit, MI 48201, USA; 12 Detroit Medical Center, Detroit, MI 48201, USA; 13 Department of Obstetrics and Gynecology, Florida International University, Miami, FL 33199, USA; 14 Center for Research and Medical Innovation, Department of Obstetrics and Gynecology, Faculty of Medicine, Universidad de Los Andes, Santiago 7620001, Chile; grice@uandes.cl; 15 UQ Centre for Clinical Research, University of Queensland, Brisbane, QLD 4072, Australia; 16 Department of Obstetrics and Gynecology, Clínica Dávila, Santiago 7620001, Chile

**Keywords:** cohort study, gestation, placental biomarkers, pre-eclampsia, risk prediction model

## Abstract

Early and innovative diagnostic strategies are required to predict the risk of developing pre-eclampsia (PE). The purpose of this study was to evaluate the performance of gingival crevicular fluid (GCF) placental alkaline phosphatase (PLAP) concentrations to correctly classify women at risk of PE. A prospectively collected, retrospectively stratified cohort study was conducted, with 412 pregnant women recruited at 11–14 weeks of gestation. Physical, obstetrical, and periodontal data were recorded. GCF and blood samples were collected for PLAP determination by ELISA assay. A multiple logistic regression classification model was developed, and the classification efficiency of the model was established. Within the study cohort, 4.3% of pregnancies developed PE. GCF-PLAP concentration was 3- to 6-fold higher than in plasma samples. GCF-PLAP concentrations and systolic blood pressure were greater in women who developed PE (*p* = 0.015 and *p* < 0.001, respectively). The performance of the multiparametric model that combines GCF-PLAP concentration and the levels of systolic blood pressure (at 11–14 weeks gestation) showed an association of systolic blood pressure and GCF-PLAP concentrations with the likelihood of developing PE (OR:1.07; 95% CI 1.01–1.11; *p* = 0.004 and OR:1.008, 95% CI 1.000–1.015; *p* = 0.034, respectively). The model had a sensitivity of 83%, a specificity of 72%, and positive and negative predictive values of 12% and 99%, respectively. The area under the receiver operating characteristic (AUC-ROC) curve was 0.77 and correctly classified 72% of PE pregnancies. In conclusion, the multivariate classification model developed may be of utility as an aid in identifying pre-symptomatic women who subsequently develop PE.

## 1. Introduction

Pre-eclampsia (PE) is defined as the new onset of hypertension and proteinuria or as the new onset of hypertension and significant end-organ dysfunction with or without proteinuria after 20 weeks of gestation in a previously normotensive woman [1,2,3,4,5]. This hypertensive disorder complicates 3–5% of all pregnancies and is one of the leading causes of maternal morbidity and mortality [5,6,7]. The severity of adverse outcomes is strongly associated with gestational age at onset. In approximately 90% of cases, PE onset after 34 weeks of gestation is associated with good health outcomes, although the mother and newborn are at increased risk of serious morbidity or mortality when compared to normotensive pregnancies [8]. Early presentation of PE (i.e., <34 weeks) is associated with poor placentation and dysfunctional spiral artery remodeling [9,10,11] and greater risk of adverse outcome, and it is associated with moderately preterm, very preterm, or extremely preterm birth [12,13,14,15,16,17]. In addition, long-term, women who developed PE are at increased risk of developing cardiovascular and renal diseases [18].

Multiple etiologies have been proposed to play a role in the pathophysiology of PE [19], principally related to an abnormal placentation and utero placental ischemia [20,21] that, in turn, are associated with an increased release of cellular debris from the trophoblast into the maternal circulation that contributes to systemic inflammation, endothelial dysfunction, and the clinical manifestation of the disease [22,23]. Until now, the only effective treatment of PE is preterm delivery of the fetus, thus removing the deleterious effects of the placenta on maternal physiology [24].

The early identification of women at risk of PE would allow for the development and evaluation of timely intervention strategies to limit immediate and long-term adverse outcomes [25,26]. Multiparametric algorithms for the identification of women at risk of developing PE have been previously reported [27,28,29,30,31]; they are based on combinations of maternal risk factors, uterine artery Doppler pulsatility, and/or different blood-borne biomarkers [32,33,34]. The development of risk assessment algorithms may increase the adoption of such testing in clinical care and improve the patient management of PE-risk pregnancies [32,35].

Recently, we identified placental molecules in gingival crevicular fluid (GCF) as a source of surrogate biomarkers of placental function [36,37,38], and the determination of such biomarkers in GCF may serve as a minimally invasive source of biomarkers for the prediction of placenta-originated diseases. Among those placental molecules, placental alkaline phosphatase (PLAP) has been linked to perinatal diseases such as preterm delivery [39,40] and PE [41,42]. PLAP is a membrane-bound glycoprotein [43] expressed by the maternal microvillous membrane of the syncytiotrophoblast [42,44,45]. The concentration of PLAP in maternal blood increases throughout gestation in normal pregnancy and has been implicated in regulating fetal/maternal metabolism, the transport of nutrients, and placental differentiation [42,44]. Moreover, in a previous case-control study, we reported significantly higher concentrations of GCF-PLAP in pregnant women with clinical PE during the third trimester compared to those with a normal pregnancy, even after adjusting for smoking status, body mass index, and periodontal diagnosis [36].

The aims of the present study are (1) to determine whether or not GCF-PLAP concentrations are increased during early pregnancy in pregnant women who subsequently develop PE and (2) to assess the classification performance of GCF-PLAP concentrations when combined with other maternal clinical parameters for the identification of pregnancies who will develop PE.

## 2. Material and Methods

### 2.1. Study Design and Participants

A prospectively collected, retrospectively stratified, observational cohort study was performed between January 2018 and March 2019 at a public health center (Hospital Sótero del Río, Santiago, Chile). Women with a singleton pregnancy less than 14 weeks of gestation and with confirmed fetal viability were invited to participate in the study. Patients under the age of 18 or with an intention of delivery at other medical centers or pregnancies with incomplete follow-up until delivery or with an unsatisfactory periodontological evaluation or GCF-PLAP measurements were excluded from the present research. The STROBE (Strengthening the Reporting of Observational Studies in Epidemiology) guidelines for reporting cohort studies adhered to the design of the study. The study was approved by the Scientific and Ethical Review Boards of the Hospital Sótero del Río and the Universidad de Los Andes, and was conducted in accordance with the Helsinki Declaration of 1973, as revised in 2003. All patients were fully informed and consented in writing to participate in this study prior to sampling and evaluation.

A detailed maternal/obstetrical history and periodontal evaluation were scheduled. Maternal systolic, diastolic, and mean arterial blood pressure, weight, and height were measured with standardized instruments. One dentist, specially trained for this study, evaluated all participants and recorded periodontal probing depth (PPD), clinical attachment level (CAL), bleeding on probing (BOP), plaque index (PI), and visible plaque accumulation, all measured along the gingival margin and recorded as the presence (+) or absence (−) of plaque and periodontal inflamed surface area (mm^2^) (PISA).

### 2.2. Definitions

In this study, pre-eclampsia (PE) was defined as a new-onset persistent blood pressure (systolic blood pressure ≥ 140 mmHg or diastolic blood pressure ≥ 90 mmHg) and proteinuria (based on a 24 h urine collection with a total protein excretion > 300 mg or a urinary spot measurement of protein-to-creatinine ratio > 0.3) or, in the absence of proteinuria, new onset hypertension with the new onset of any of the following: thrombocytopenia (platelets < 100 × 10^9^/L), renal insufficiency (serum creatinine > 1.1 mg/dL or doubling creatinine in the absence of other renal disease), impaired liver function (elevated blood concentrations of liver transaminases to twice normal concentration), pulmonary edema, and unexplained new-onset headache unresponsive to medications or visual symptoms after 20 weeks of gestation, according to the American College of Obstetricians and Gynecologists (ACOG 2018).

Periodontitis was defined according to the classifications established by the 2017 World Workshop [46,47]: (1) interdental clinical attachment level (CAL) detectable in ≥2 non-adjacent teeth or (2) buccal or oral CAL ≥ 3 mm with pocketing > 3 mm detectable in ≥2 teeth. Gingivitis was defined in subjects who did not exhibit a periodontal probing depth (PPD) ≥ 3 mm, who were without CAL, and who had positive bleeding upon probing (BOP) in ≥10% of probe sites. Gingival health was defined as <10% BOP sites, with a PPD ≤ 3 mm [48].

### 2.3. Gingival Crevicular Fluid Sample Collection and Elution Protocol

The collection of GCF samples was performed between 11 and 14 weeks of gestation. Samples were obtained from four periodontal sulcus/pockets (1 × quadrant) at the most affected periodontal site, representative of the periodontal diagnosis of the patient, and then the PerioPaper^®^ strips (ProFlow, Amityville, NY, USA) were pooled to make one sample. The sampling tooth was isolated with a cotton roll, the supragingival plaque was slightly removed with curettes, without contacting the gingival margin, and then gently dried with an air syringe. GCF was collected by using paper strips. The strips were placed into the sulci/pocket until mild resistance was sensed and left in place for 30 s. Strips contaminated by saliva or blood were excluded from the study. The collected pooled strips were subsequently eluted in 160 µL of elution buffer with added 0.5 M Tris-HCl, pH 7.5, NaCl_2_ M, 250 mM CaCl_2,_ and Triton X100 at 25% concentration, adding EDTA-free protease inhibitor cocktail (Complete^®^, Mini, EDTA-free protease Inhibitor Cocktail, *EASY*pack, Roche, Basel, Switzerland). Then, the samples were vortexed for 30 s, incubated for 30 min on ice, and centrifuged at 4 °C for 5 min at 12,000× *g*. The supernatant was kept on ice and transferred into a new 1.5 mL Eppendorf tube and the process was repeated. The final 320 µL of the eluted sample was stored at −80 °C until PLAP analysis.

### 2.4. Blood Samples

Fasting blood samples were collected by venipuncture into EDTA-containing tubes between 8:00 am and 10:00 am and then were separated by centrifugation at 1000× *g* for 15 min at 4 °C. All the samples were frozen and stored at −80 °C until ELISA analysis.

### 2.5. ELISA Assays

PLAP concentrations were quantified by using the commercially available Placental Alkaline Phosphatase ELISA kit (catalog no. MBS701995; MyBiosource, San Diego, CA, USA). The sandwich ELISA used mouse monoclonal antibodies raised against full-length and partial-length recombinant human placental alkaline phosphatase (P05187) for the capture and detection antibodies, respectively, and has been reported to have no significant cross-reactivities (mybiosource.com (accessed on 30 March 2021)). Previously, this ELISA has been used to quantify human PLAP in early pregnancy plasma [49]. The sensitivity was 0.39 ng/mL, the intra-assay coefficient of variation (CV) was <8%, and the inter-assay precision CV was <10%. The samples were read at a wavelength of 450 nm in an automatic ELISA plate reader (CM Sunrise™ 350–700 nm, Tecan US, Inc., Seestrasse, Switzerland).

### 2.6. Sample Size Calculation

The estimated sample size was calculated based on previous observations of differences between GCF-PLAP concentrations in women with PE and normotensive pregnant women (2044 ± 217 and 1880 ± 82 pg/mL; mean ± standard deviation, respectively) [36]. To test the hypothesis of mean differences in GCF-PLAP between patients affected by PE and heathy controls, a minimum cohort size of 406 pregnant women was calculated based on the following assumptions: a 6.4% prevalence of PE in the entire cohort; a significance level of 5%; a power of 80%; a two-sided test; and a loss-to-follow-up of 5%.

### 2.7. Statistical Analyses

Shapiro–Wilk tests were used to assess data normality. Non-parametric tests were used to assess statistical differences. Comparisons between proportions were performed with a chi-squared or Fisher’s exact test, and the Mann–Whitney U test was used to compare continuous variables. The association strength was assessed by using a multiple logistic regression model that was adjusted by systolic blood pressure and PLAP-GCF concentrations. Area under the receiver operating characteristic curves (AUC-ROCs) summarized the performance of PLAP. Goodness of fit and internal validation of the model were assessed by using the Bayesian Information Criterion (BIC) and bootstrapping. The statistical analysis was performed by using a commercially available software package (STATA software, StataCorp version 14.1, Lakeway Drive College Station, TX, USA). A *p*-value < 0.05 was considered statistically significant.

## 3. Results

**Figure 1:**
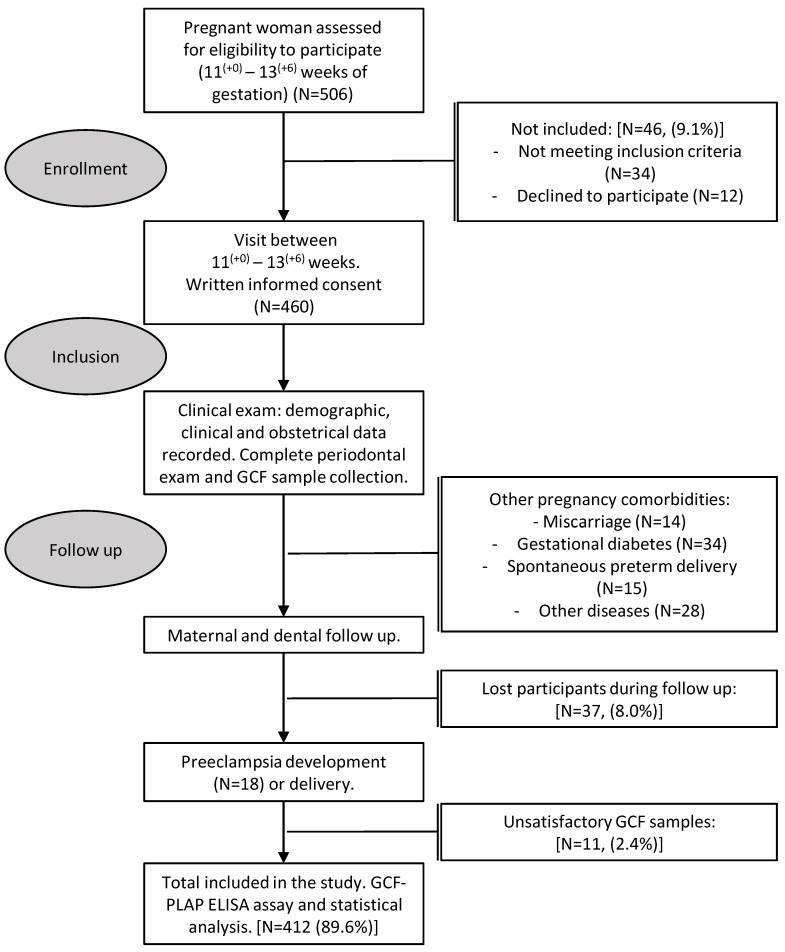
Flow chart of the study population.

A study design flowchart is presented in Figure 1. Of the 460 singleton pregnant women recruited into this study, 423 (92%) completed the follow-up until delivery. In 11 cases (2.6%), GCF-PLAP samples were unsatisfactory for analysis and were excluded from the study; therefore, 412 cases (89.6%) were available for analysis.

The baseline characteristics of the study population are summarized in Table 1. Of the 412 pregnant women recruited and followed throughout pregnancy, 18 of them subsequently developed PE (4.3%), and five (1.2%) required delivery before 37 weeks of gestation. Systolic blood pressure (112 mmHg, interquartile range (IQR): 6, *p*-value < 0.001), diastolic blood pressure (76 mmHg, IQR: 10, *p*-value = 0.007), and median arterial blood pressure (89 mmHg, IQR: 9, *p*-value = 0.006), measured during early pregnancy (11–14 weeks of gestation), were significantly higher in women who developed PE when compared to controls. Of them, 30.1% of pregnancies were primiparas and 69.9% multiparas, and the rate of prior cesarean delivery was 47.9%. Previous antecedents of PE were present in 1.6% of the cohort, and the rates of other comorbidities presented were as follows: miscarriage 3.39%, gestational diabetes 8.25%, spontaneous preterm delivery 3.64%, and other diseases 6.79%.

No statistically significant differences in periodontal parameters were identified between patients who developed PE and controls, and no statistically significant association was identified between periodontal clinical diagnosis and the subsequent development of PE (*p*-value = 0.617). The median maternal GCF-PLAP concentration was 63.7 (interquartile range (IQR): 88.9) pg/mL in healthy patients (12,3%), 46.6 (IQR: 47.2) pg/mL in patients with gingivitis (28.5%), and 42.4 (IQR: 46.7), 41.1 (IQR: 51.8), 34.7 (IQR: 46.88) pg/mL at periodontitis stage I (33.1%), stage II–III (16.1%) and stage IV (10%), respectively, without statistically significant differences among them (*p*-value = 0.407).

**Figure 2:**
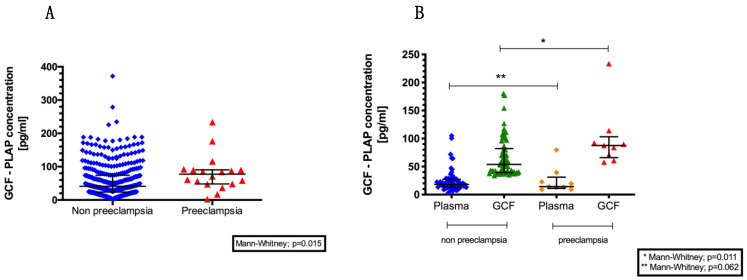
(**A**) Placental alkaline phosphatase (PLAP) concentrations (pg/mL) in gingival crevicular fluid (GCF) in pregnancy according to the presence or absence of pre-eclampsia. (**B**) Plasma and GCF-PLAP concentrations at 11–14 weeks of gestation in women with and without pre-eclampsia. GCF, gingival crevicular fluid; PLAP, placental alkaline phosphatase.

GCF-PLAP concentrations at 11–14 weeks were compared between patients who subsequently developed PE and controls. The median maternal GCF-PLAP concentration was significantly higher in the PE group (77.5 pg/mL (IQR: 41.5) vs. 41.3 pg/mL (IQR: 50.1), (*p*-value = 0.015) (Figure 2A). In addition, PLAP concentrations were also measured in paired plasma and GCF samples from 80 women from the same cohort. The median plasma PLAP concentrations were 24.2 pg/mL (IQR: 2.5) and 24.6 pg/mL (IQR: 7.6) in the control and PE groups, respectively. In the paired GCF samples, median PLAP concentrations were 66.1 pg/mL (IQR: 4.3) and 99 pg/mL (IQR: 17.8) in the control and PE groups, respectively (*p*-value = 0.011) (Figure 2B). The observed amount of GCF-PLAP was 3- to 6-fold higher than in plasma samples.

**Figure 3:**
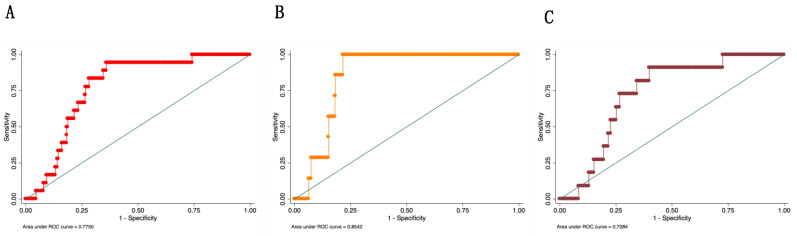
(**A**) Area under the receiver operating characteristic curve (AUC-ROC) of PLAP-GCF concentration and systolic blood pressure at 11–14 weeks of gestation versus the development of pre-eclampsia. (**B**) Area under the receiver operating characteristic curve (AUC-ROC) of the concentration of PLAP-GCF and systolic blood pressure versus the development of preterm pre-eclampsia. (**C**) Area under the receiver operating characteristic curve (AUC-ROC) of the concentration of PLAP-GCF and systolic blood pressure at 11–14 weeks gestation versus pre-eclampsia > 37 weeks of gestation. GCF, gingival crevicular fluid; PLAP, placental alkaline phosphatase.

The performance of the multiparametric model that combines GCF-PLAP concentration and the levels of systolic blood pressure (at 11–14 weeks gestation) showed an association of systolic blood pressure and GCF-PLAP concentrations with the likelihood of developing PE (OR:1.07; 95% CI 1.01–1.11; *p* = 0.004 and OR:1.008, 95% CI 1.000–1.015; *p* = 0.034, respectively) (Table 2). The results of the bootstrap analysis were similar to those observed in the logistic regression model (Table 2). The GCF-PLAP concentration combined with systolic blood pressure at 11–14 weeks of gestation was found to be a good predictor of PE, with a specificity of 72%, a sensitivity of 83%, a positive predictive value of 12%, and a negative predictive value of 99%. The positive likelihood ratio was 2.9, and the negative likelihood ratio was 0.3. The model correctly classified 72% of the women who developed PE. The AUC for GCF-PLAP concentrations alone at 11–14 weeks of gestation was 0.67, for systolic blood pressure, 0.74, and for GCF-PLAP concentrations and systolic blood pressure, 0.77 (95% CI: 0.70–0.85) (Figure 3A). In the sub-analysis, dividing the PE pregnancies into preterm PE (≤37 weeks) and term PE (≥37 weeks), the observed AUC was 0.85 (95% CI: 0.81–0.93) for preterm PE and 0.72 (95% CI: 0.58–0.82) for term PE (Figure 3B,C, respectively). All five cases (100%) of preterm PE observed in the current study were correctly classified by the model.

## 4. Discussion

To our knowledge, this is the first cohort study evaluating the classification efficiency of GCF-PLAP concentrations for early-pregnancy risk assessment of PE. The data obtained support the hypothesis that GCF-PLAP concentrations are significantly increased in pre-symptomatic women who subsequently develop PE. These data are consistent with and extend our previous observations [36].

In addition, GCF-PLAP concentrations were significantly greater than those measured in paired plasma samples. Specifically, women who developed PE presented with 3- to 6-fold greater GCF-PLAP concentrations than those measured in matched plasma samples. Our findings support the hypothesis that potential biomarkers of obstetric diseases can be concentrated in GCF, highlighting the opportunity to use placental biomarkers measured in GCF to improve the performance of risk assessment models of PE. Currently available data, however, do not provide insight into the mechanism(s) by which PLAP may concentrate in GCF. Possible mechanisms may include PLAP-dependent targeting of sites of inflammation and its association with complementary proteins, or the selective accumulation of PLAP-containing extracellular vesicles at such sites.

Finally, GCF-PLAP concentrations, measured at 11–14 weeks of gestation, were predictive of PE when combined with maternal systolic blood pressure in a multivariate predictive model. The performance of the classification model was satisfactory, with an observed AUC of 0.77 (95% CI: 0.70–0.85) for all PE cases, 0.85 (95% CI: 0.81–0.93) for preterm PE, and 0.72 (95% CI: 0.58–0.82) for term PE. A caveat, when interpreting the data obtained, is that the study was neither designed nor powered to differentiate between putative subtypes of pre-eclampsia but rather to identify pre-symptomatic women at risk of developing PE.

Increased plasma PLAP concentrations are significantly increased in pregnant women with hypertensive disorders [44,50] and associated with PE as a result of placental dysfunction, and also might represent an informative biomarker of the syncytiotrophoblast function [45,51]. During pregnancy, syncytiotrophoblastic debris is normally shed into the maternal circulation; however, shedding is significantly increased in pregnancies complicated by PE [45,51]. Furthermore, replenishment of the syncytiotrophoblast is intense, complicated by necrosis and aponecrosis with increased liberation into the circulation of syncytiotrophoblast-derived particles in PE [19,50,52,53]. In line with our result, syncytiotrophoblast-derived particles, such as PLAP, can reach the gingival sulcus during early pregnancy and can be detected in the GCF. In fact, its concentrations are informative of the risk of developing PE and may be potentially used in future multiparametric algorithms for the prediction of the disease.

The most recently developed multivariate algorithms, using different combinations of maternal risk factors, biophysical variables such as mean maternal blood pressure, uterine artery pulsatility index, and maternal plasmatic biomarkers such as placental growth factor and/or pregnancy-associated plasma protein A, at 11–14 weeks of gestation, have consistently demonstrated detection rates of preterm PE of over nearly 70% at a false-positive rate of 10% [32,35,54]. In fact, plasmatic individuals’ biomarkers, which include a disintegrin and metalloproteinase 12 (ADAM-12), inhibin-A, pregnancy-associated plasma protein A (PAPP-A) and placental protein 13 (PP-13), have shown a low predictive value for PE during the first trimester of pregnancy [55].

The results obtained in this study further support the use of multiparametric algorithms for improving the prediction of PE. The model reported herein is based on the measurement of a single biomarker in GCF and systolic blood pressure; therefore, it is non-invasive and inexpensive. Moreover, it displays potential clinical utility in identifying women during early pregnancy who are at increased risk of developing PE, especially preterm PE, and, as such, warrants further clinical evaluation.

Regarding the link between periodontal disease and the risk of PE, our study did not confirm the association between periodontal diagnoses and PE that had been previously described in the literature [56,57,58,59,60]. These results, however, should be interpreted with caution given that the present study was powered to determine the association between GCF-PLAP concentrations and PE but not the association between PE and periodontal diagnosis. In addition, in our study, periodontal disease was assessed during early pregnancy and in a young population of pregnant women. It is known that periodontal disease usually worsens during pregnancy [61,62,63] and that its evaluation at a later stage of pregnancy may be more related to the development of PE.

In summary, the development of more innovative diagnostic tests that allow for the early identification of women at risk of developing PE is a recognized clinical need. The data obtained in this study are consistent with the hypothesis that the accumulation of placental molecules within GCF during early pregnancy is informative of the risk of developing PE and could be surrogate markers of placental function. The practical translation of these data into a clinical setting requires further validation to determine its clinical implications. This transition will be facilitated in countries where periodontal evaluations are already part of routine pregnancy healthcare delivery (e.g., Chile). Future studies are required to confirm our results and to address the predictive capabilities of CGF-PLAP concentration alone and/or in combination with more variables and risk factors, such as uterine artery Doppler, to further increase the performance of the algorithm.

## Figures and Tables

**Table 1:** Clinical, demographic, and periodontal descriptions of pregnant women at 11–14 weeks of gestation.

Variable	No Pre-Eclampsia	Pre-Eclampsia
	(*n* = 394)	(*n* = 18)
Age (years)	26 (8) 18–41	26 (9) 19–38
Weight (kg)	68 (17) 40–128	68 (25) 36–112
Height (mts)	158 (7) 129–175	159 (8) 147–170
Body mass index (kg/m^2^)	27 (7.1) 16.9–49	28 (8.0) 15.4–39.2
Systolic blood pressure (mmHg)	104 (10) 80–142	112 (6) 100–124
Diastolic blood pressure (mmHg)	62 (8) 40–92	67 (6) 52–80
Mean arterial pressure (mmHg)	84 (12) 60–113	87.5 (12) 78–101
Maternal active smoking (%)	72 (18.3)	5 (27.8)
First-trimester glycaemia (mg/dL)	86 (8) 66–216	89 (11) 79–95
Fasting glycaemia (mg/dL) (second trimester)	83 (3) 60–141	90 (13) 75–93
Oral glucose tolerance test (second trimester)	103 (8) 65–201	113 (46) 69–153
Plaque index (PI) (%)	67 (51) 0–100	77 (37) 14–100
Bleeding on probing (BOP) (%)	56 (43) 2–100	58 (38) 2–100
Periodontal probing depth (PPD) (mm)	2.6 (0.6) 1.4–4.4	2.6 (0.7) 1.5–4
Clinical attachment level (CAL) (mm)	1.9 (0.7) 0.9–5.3	2 (0.7) 1.3–4.2
Periodontal probing depth pockets ≥ 3 mm (%)	12.2 (18.3) 0–70.8	10.2 (18.8) 0–55.9
Periodontal inflamed surface area (mm^2^)	777.6 (769.8) 10.7–2604	791.6 (589.7) 11.7–2515

Results are expressed as median (interquartile range) and minimum and maximum values or frequencies and (%). *p* = < 0.001; *p* = 0.007; *p* = 0.006.

**Table 2:** Association between GCF-PLAP concentration and systolic blood pressure at 11–14 weeks of gestation, according to the presence or absence of pre-eclampsia: multiple regression logistic models (A) and bootstrap estimation of the multiple logistic regression model (B).

**Primary multiple logistic regression model**					
Pre-eclampsia	Odds ratio	Standard error	*p*-value		95% CI
GCF-PLAP concentration:	1.008	0.0038	0.034		(1.000–1.015)
Systolic blood pressure:	1.066	0.023	0.004		(1.020–1.11)
**Bootstrap estimation of the multiple logistic regression model**					
Coefficients	Mean	Standard error		95% CI Bootstrap	
Intercept	−10.784	1.617		(−13.956–−7.613)	
GCF-PLAP (pg/mL)	0.008	0.003		(0.000–0.015)	
Systolic blood pressure	0.065	0.014		(0.037–0.094)	

CI, confidence interval; GCF, gingival crevicular fluid; PLAP, placental alkaline phosphatase.

## Data Availability

Not applicable.
